# Porous Functionalized Covalent-Triazine Frameworks for Enhanced Adsorption Toward Polysulfides in Li-S Batteries and Organic Dyes

**DOI:** 10.3389/fchem.2020.584204

**Published:** 2020-12-02

**Authors:** Qianhui Liu, Shuhao Yang, Hlib Repich, Yixuan Zhai, Xiaosa Xu, Yeru Liang, Hejun Li, Hongqiang Wang, Fei Xu

**Affiliations:** ^1^State Key Laboratory of Solidification Processing, Center for Nano Energy Materials, School of Materials Science and Engineering, Northwestern Polytechnical University, and Shaanxi Joint Laboratory of Graphene (NPU), Xi'an, China; ^2^College of Materials and Energy, South China Agricultural University, Guangzhou, China

**Keywords:** covalent triazine-based frameworks, pore structure, functionalization, lithium- sulfur batteries, organic dye adsorption

## Abstract

The incorporation of functional building blocks to construct functionalized and highly porous covalent triazine frameworks (CTFs) is essential to the emerging adsorptive-involved field. Herein, a series of amide functionalized CTFs (CTF-PO71) have been synthesized using a bottom-up strategy in which pigment PO71 with an amide group is employed as a monomer under ionothermal conditions with ZnCl_2_ as the solvent and catalyst. The pore structure can be controlled by the amount of ZnCl_2_ to monomer ratio. Benefitting from the highly porous structure and amide functionalities, CTF-PO71, as a sulfur cathode host, simultaneously demonstrates physical confinement and chemical anchoring of sulfur species, thus leading to superior capacity, cycling stability, and rate capability in comparison to unfunctionalized CTF. Meanwhile, as an adsorbent of organic dye molecules, CTF-PO71 was demonstrated to exhibit strong chemical interactions with dye molecules, facilitating adsorption kinetics and thereby promoting the adsorption rate and capacity. Furthermore, the dynamic adsorption experiments of organic dyes from solutions showed selectivity/priority of CTF-PO71s for specific dye molecules.

## Introduction

Porous Organic Frameworks (POFs) possess unique structural features, such as open and permanent porosity, high surface area, and flexible molecular design of pore structures and functionalities. POFs have thus drawn much attention due to their innovative synthesis and various potential applications (DeBlase et al., 2013; Zeng et al., 2015; Banerjee et al., 2017; Lin et al., 2017; Hynek et al., 2018; Lu Q. et al., 2018; Wang L. et al., 2018; Wang Z. et al., 2018). Covalent Triazine Frameworks (CTFs) are a class of important POFs, pioneered by Thomas and Kuhn et al. through ionothermal synthesis (Kuhn et al., 2008). CTFs are promising due to their highly specific surface area, nitrogen-rich skeleton, good thermal stability, and conductive properties (both partial and full). Since then, many CTFs have been developed and found to have promising potential for a wide range of applications, including gas adsorption, energy storage, medicine delivery, and catalysis (Tao et al., 2014; Fu et al., 2018; Du et al., 2019; Lu et al., 2019). One of the key elements that determine the properties of CTFs on these capture-based applications could be pore structures, including specific surface area and pore size, of which the micropores (< 1 nm) contribute to the capture of small guest molecules/ions, whereas meso- and/or macropores facilitate their diffusion (Lu Y. et al., 2018). Key elements also include the functionalities, which could be built in the pore skeletons using functionalized monomers or through post-modification with the aim to bringing active sites to specific reactions and effectively tailor the properties of CTFs (Sun et al., 2018; Wang Z. et al., 2018; Wu et al., 2019). The construction of novel CTFs with optimized pore structures and functionalized active sites is thus of critical importance to realizing their application-oriented design (Kuhn et al., 2008; Kamiya et al., 2014; Li J. et al., 2018; Zhou et al., 2018).

Recently, POFs have drawn attention as a new class of cathode host materials for Li-S batteries due to their high porosity and predesignable frameworks (Wang Z. et al., 2014; Xu et al., 2018, 2019b; Wang et al., 2019). The semiconductive properties of CTFs in particular render them more intriguing compared with other POF-based sulfur hosts (Talapaneni et al., 2016; Xu et al., 2017b; Yang et al., 2019; Troschke et al., 2020). Essentially, the use of POFs as sulfur hosts involves capture/adsorption processes toward soluble intermediate polysulfides, which is the fundamental reason for inducing the so-called shuttle effect during the sulfur redox reaction, thus deteriorating the performance of lithium-sulfur (Li-S) batteries. An ideal POFs sulfur host should possess the following structural features: (1) semi or fully conductive frameworks for improving electrical conductivity; (2) tremendous nanopores for suppressing the diffusive loss of polysulfides by physical entrapment; and (3) suitable framework functionalities for strong chemical anchoring polar polysulfides. It is therefore highly desirable to explore highly porous and functionalized CTFs in Li-S batteries.

In addition to sulfur host materials, removal of toxic organic dyes from wastewater using porous materials also involves an adsorptive process, and it is an emerging task where effective adsorbent materials are highly desirable. POF-based adsorbents not only possess high surface areas and tailored pore sizes for physical adsorption but also show flexible molecular designs with targeted functionalities to trigger a stronger chemical interaction with dye molecules, thereby promoting the adsorption performance. The development of POFs with high surface areas, suitable pore sizes, and site-specific functionalities is consequently promising for advanced adsorbents (Byun et al., 2016; Konavarapu and Biradha, 2018; Wu et al., 2019).

Herein, we have prepared a series of amide-functionalized CTFs (CTF-PO71s) with well-developed porosity through a bottom-up strategy, utilizing an amide-bearing PO71 monomer under ionothermal conditions of ZnCl_2_. We studied the contribution of pore structure and amide-based functionalities of CTFs to adsorption-based applications by applying the CTF-PO71 as a sulfur cathode host for Li-S batteries and as an adsorbent for aqueous organic dye molecule removal and selective adsorption. On the basis of the obtained results, we have also discussed the effect of physical capture provided by nanopores and the chemical interaction attributed to the amide groups, and we have made a comparison of the properties with unfunctionalized CTF materials (Kuhn et al., 2008) (abbreviated as CTF in the following) toward those applications, thus establishing the coordination of pore structure and functional active sites on the performance of the capture-based process.

## Materials and Methods

### Preparation of CTF-PO71

In a typical process (Lu Y. et al., 2018), pigment orange-71 (PO71, 0.2 g, 0.59 mmol) and ZnCl_2_ (1 g, 7.35 mmol) were placed into a glass ampoule in a glove box. The ampoule was then flame sealed under vacuum and heated in a muffle oven at a rate of 1°C min^−1^ to 400°C and kept at this temperature for 40 h. After cooling, the product was soaked and stirred in HCl solution (1 M) for 24 h to wash out the metal ions. Finally, the product was washed with deionized water, ethanol, and THF and dried at 80°C for 8 h. The other CTF-PO71 samples were fabricated through the same route except for the mass ratios of PO71 to ZnCl_2_, i.e., 1:1 for CTF-PO71-1 and 1:10 for CTF-PO71-10.

### Li-S Cell Assembly and Electrochemical Measurements

The electrodes were fabricated using a slurry-coating method. The active material, conductive additive (Super P), and binder polyvinylidene fluoride binder (PVDF) were mixed in a weight ratio of 7:2:1 in N-methyl-2-pyrrolidinone (NMP) and transformed into slurries using ball milling. The slurries were coated on carbon-coated aluminum foil and dried under 60°C for 8 h. The as-prepared electrode was cut into round disks with a diameter of 12 mm and further dried under 60°C in a vacuum oven for 12 h before use. The electrochemical measurements were carried out with CR2032-type coin cell. The CTF-PO71/S or CTF/S electrodes were applied as working electrodes, and Li foil was used as a counter and reference electrode. The electrolyte was a solution of 1 M LiTFSI in a mixture of 1,3-dioxolane (DOL) and 1,2-dimethoxyethane (DME) (1:1 by volume) with 0.5 wt% LiNO_3_ additive, and the added amount in each cell was 30 μL. The Celgard 2500 membrane was used as a separator, and the cells were assembled in a glove box in an argon atmosphere. The sulfur loading was 0.45~0.55 mg cm^−2^ on each electrode. The electrochemical performance of the electrodes was tested at room temperature. Discharge/charge performance of the cells was measured at the LAND electrochemical workstation. The cyclic voltammogram (CV) performance with a fixed potential range of 1.7~2.8 V (vs. Li^+^/Li) was tested on the CHI660 electrochemical station.

### Organic Dye Adsorption

A total of 50 mL of Methylene blue (MB) aqueous solution with a concentration of 30 mg L^−1^ was prepared and transferred into an Erlenmeyer flask and kept at 29°C in a water-bath thermostatic oscillator. A total of 35 mg of adsorbent (CTF, CTF-PO71s) was added to the organic dye solution to form a homogeneous suspension under reciprocated shake with a rate of 150 rpm. The after-adsorption solutions at various adsorption times were aspirated by syringes, and the concentrations of the dye were measured using UV-Vis spectrophotometer. Firstly, the maximum absorption wavelength of the organic dye was determined by measuring the absorbance of visible light (400~760 nm). Then absorbances of a series of dye solutions with known concentrations were measured at the maximum absorption wavelength to obtain a series of absorbance values. A calibration curve of concentration and absorbance of the organic dye was thus established. The concentration and absorbance satisfy the linear function in a limited range at low concentration. The concentrations of the dyes times were then determined after different adsorption, and the concentration vs. adsorption time isotherm was thus built. The adsorption capacity of the adsorbent (CTF-PO71s, CTF) on dye molecules (MB, R6G) could be calculated using the following equation:

$$\begin{array}{l} q_{t}=\frac{\left(C_{0}-C_{t}\right)V}{m} \end{array}$$

where *q*_*t*_ stands for the adsorption capacity under a certain time (g^−1^), *V* refers to the volume of organic dye solution (L), and *m* is the mass of adsorbent (mg).

## Result and Discussion

### Materials Characterization

**Figure 1:**
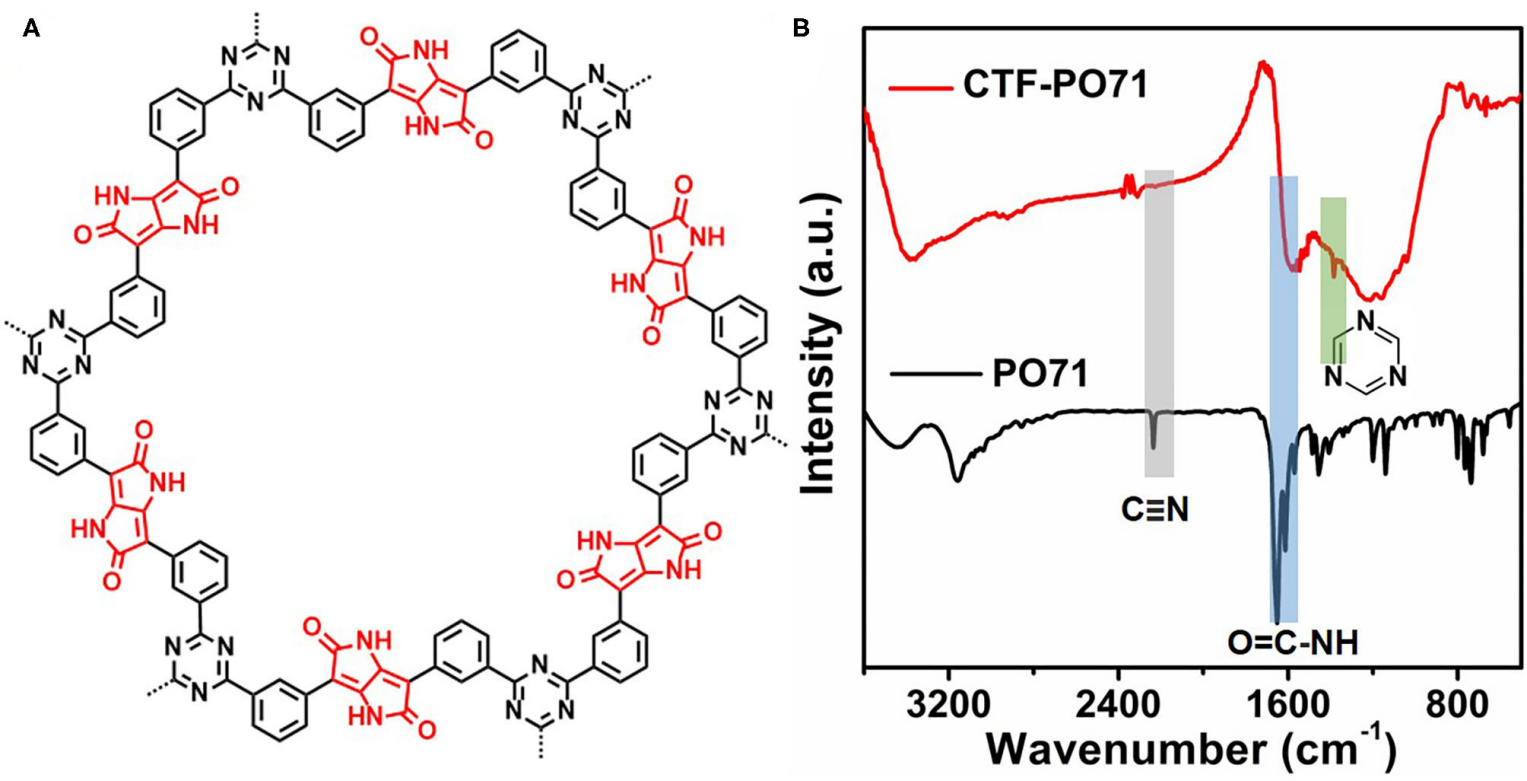
**(A)** Chemical structure of CTF-PO71. **(B)** FT-IR spectra of CTF-PO71 and PO71.

**Figure 2:**
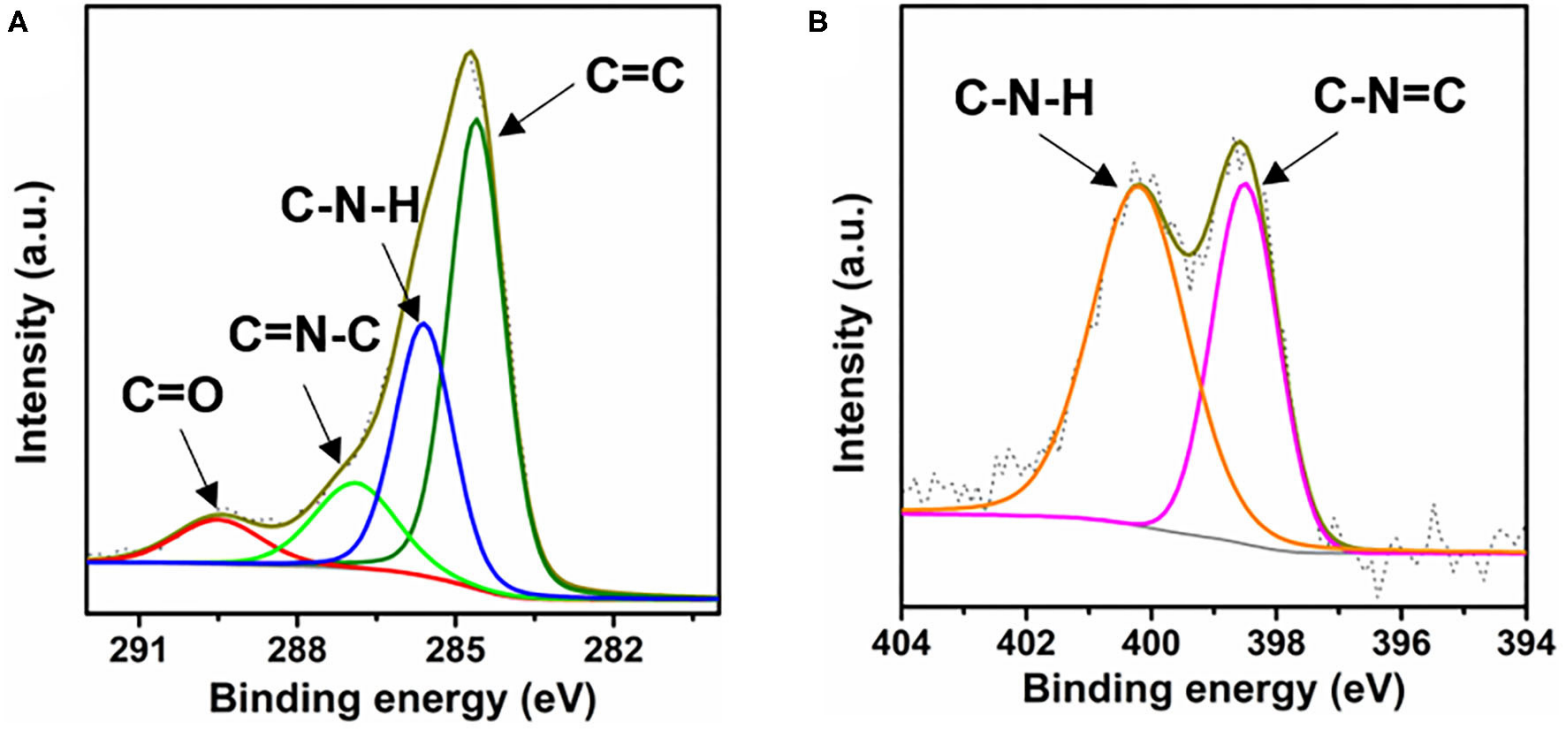
XPS spectra of **(A)** C1s and **(B)** N1s of CTF-PO71.

**Figure 3:**
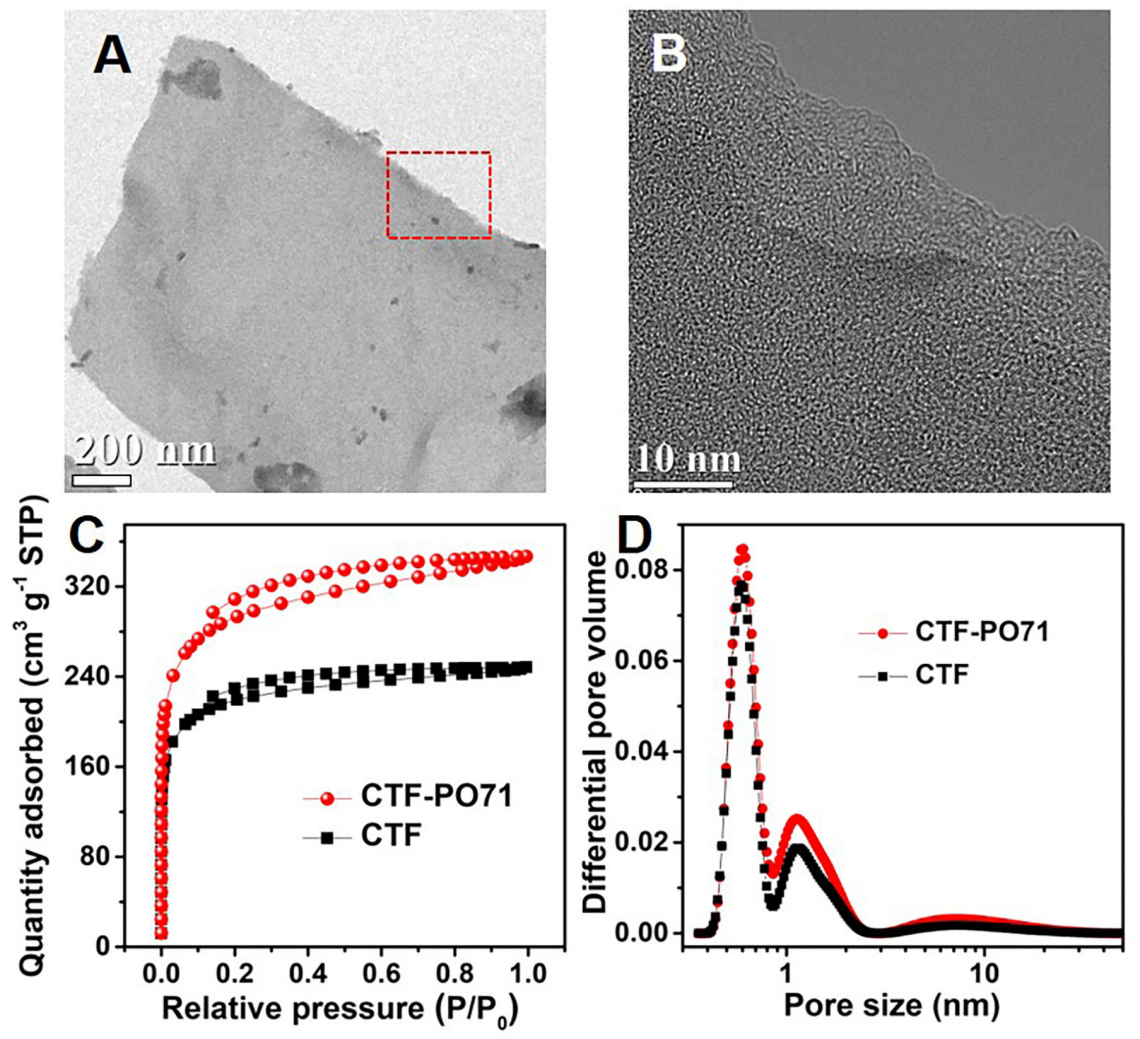
**(A,B)** TEM images of CTF-PO71. **(C)** Nitrogen adsorption–desorption isotherms, and **(D)** NLDFT pore size distribution curves of CTF-PO71 and CTF.

The CTF-PO71s were synthesized through trimerization of PO71 under ionothermal conditions (Lu Y. et al., 2018), where ZnCl_2_ serves as both the catalyst and solvent (Figure 1A). In the following, the CTF-PO71 refers to the sample obtained with PO71 to ZnCl_2_ mass ratio of 1:5 unless otherwise mentioned. The CTF-PO71s and CTF were characterized by a variety of methods in order to confirm their structure. Figure 1B shows the Fourier transform infrared (FT-IR) spectra of CTF-PO71 and PO71. The disappearance of the -CN stretching band at 2235 cm^−1^ in the CTF-PO71 in comparison to PO71 demonstrates the completion of a trimerization reaction in agreement with CTF with 1,4-dicyanobenzene as a monomer (Supplementary Figure 1). The amide-related bands at around 3375 and 1600 cm^−1^ are also observed in CTF-PO71, indicating the presence of a functional amide group. The absorption band at 1530 ~ 1580 cm^−1^, which is characteristic for a s-triazine ring, might be overlapped with the broad peak of C=O, and the stretching vibration at 1384 cm^−1^ also demonstrates the presence of s-triazine. To further verify the chemical structure of CTF-PO71, X-ray photoelectron spectroscopy (XPS) was applied. High resolution XPS C1s spectrum (Figure 2A) shows four main contributions, where the peaks at 284.6, 286.9 eV correspond to C atoms from benzene and triazine rings, respectively, and the other two peaks at 285.6 and 289.5 eV are assigned to C-N-H and C=O, respectively. In the N1s spectrum (Figure 2B), two peaks at 398.5 eV and 400.2 eV can be attributed to the N atoms in triazine rings and the amide group, respectively. Meanwhile, the peak at 532.9 eV in O1s spectrum could be assigned to the C=O from amide group (Supplementary Figure 2). The powder X-Ray diffraction (PXRD) patterns of CTF-PO71s and CTF (Supplementary Figure 3) all show a broad peak around 23°, revealing their amorphous structures (Tao et al., 2014). Field-Emission Scanning Electron Microscopy (FE-SEM) images reveal that CTF-PO71 shows bulky particles with some tiny particles adhered on the smooth surface (Supplementary Figure 4). Transmission Electron Microscopy (TEM) image shows that CTF-PO71 has a layered structure (Figure 3A). A high-resolution TEM image reveals that the layered sheets are microporous (Figure 3B).

The porous structure of CTF-PO71 was further investigated by N_2_ adsorption–desorption measurements at 77 K (Figure 3C). The CTF-PO71 shows typical I type isotherm curve with a sharp increment of uptake at low relative pressure region, indicating the presence of great amount of micropores, which agrees with the observation in magnified TEM image. In contrast, CTF exhibits less adsorption amount, manifesting fewer micropores. Consequently, the specific surface area of CTF-PO71 calculated by Brunauer–Emmett–Teller (BET) method is 1001 m^2^ g^−1^, which is greater than that of CTF (746 m^2^ g^−1^). The pore size distribution curve (Figure 3D) calculated by nonlocal density function theory (NLDFT) further verifies the microporous characteristics with pore sizes of ~0.61 nm, which might result from partial carbonization by ZnCl_2_ during the high-temperature process. This highly microporous structure demonstrates a potential of the CTF-PO71 for small guest molecules capture. There is another less intense peak at 1.13 nm, which has resulted from the CTF hexagonal skeleton.

By further tuning the weight ratio of PO71 and ZnCl_2_ from 1:1 to 1:10, CTF-PO71-1 and CTF-PO71-10 were obtained, respectively. CTF-PO71-10 exhibits a significantly higher BET specific surface area of 1519 m^2^ g^−1^ (Supplementary Figure 5). This might be caused by the use of high amount of ZnCl_2_ that seriously degraded the network of polymer by activation, leading to the partial carbonization of the sample (Mukherjee et al., 2019). The largest mesopore ratio for CTF-PO71-10 compared with that of other two samples also proves partial activation and destruction of the molecular structure (Supplementary Table 1). On the other side, CTF-PO71-1 also shows a slightly higher specific surface area than CTF-PO71, which may result from more regular and better retention of polymeric structure, providing larger amounts of micropores that contribute to the surface area. XPS spectra of all these three samples further verified their chemical structure (Supplementary Table 2). The value of N to C of as-obtained CTF-PO71s samples deviates from theoretical value of 0.2, and by increasing the mass ratio of ZnCl_2_ to PO71, the value decreases gradually from 0.11 to 0.06, further confirming that excess of ZnCl_2_ causes a partial collapse of polymer structures. The contents of O among all three samples show an obvious higher value compared with that from the theoretical model, which may be due to the adsorbed oxygen species in the pores. Considering the low cost of the raw materials and the simple reaction procedure, there is a great opportunity for scaling up production.

### Sulfur Host for Lithium-Sulfur Batteries

Lithium-sulfur batteries are considered as promising next generation rechargeable batteries with high energy densities resulting from the high theoretical capacity of 1675 mAh g^−1^ of sulfur (Wang Z. et al., 2014; Talapaneni et al., 2016; Xu et al., 2017a). One of the critical obstacles is the shutting of polysulfides, which are soluble in electrolytes, giving rise to diffusive loss of sulfur and rapid capacity fading and low sulfur utilization. Covalent triazine frameworks (CTFs) show potential as sulfur hosts due to their high porosity, polar-nitrogen-rich framework, and stable chemical structure (Liao et al., 2014; Talapaneni et al., 2016). By incorporation of specific functional groups that are able to interact with polysulfides chemically, functional CTFs with high surface areas are expected to achieve efficient immobilization of polysulfides through physical confinement and chemical interaction approaches, thereby further boosting the performance of lithium-sulfur batteries. In this context, the present CTF-PO71 is advantageous in consideration of its amide groups and developed pore structures, as elucidated below.

**Figure 4:**
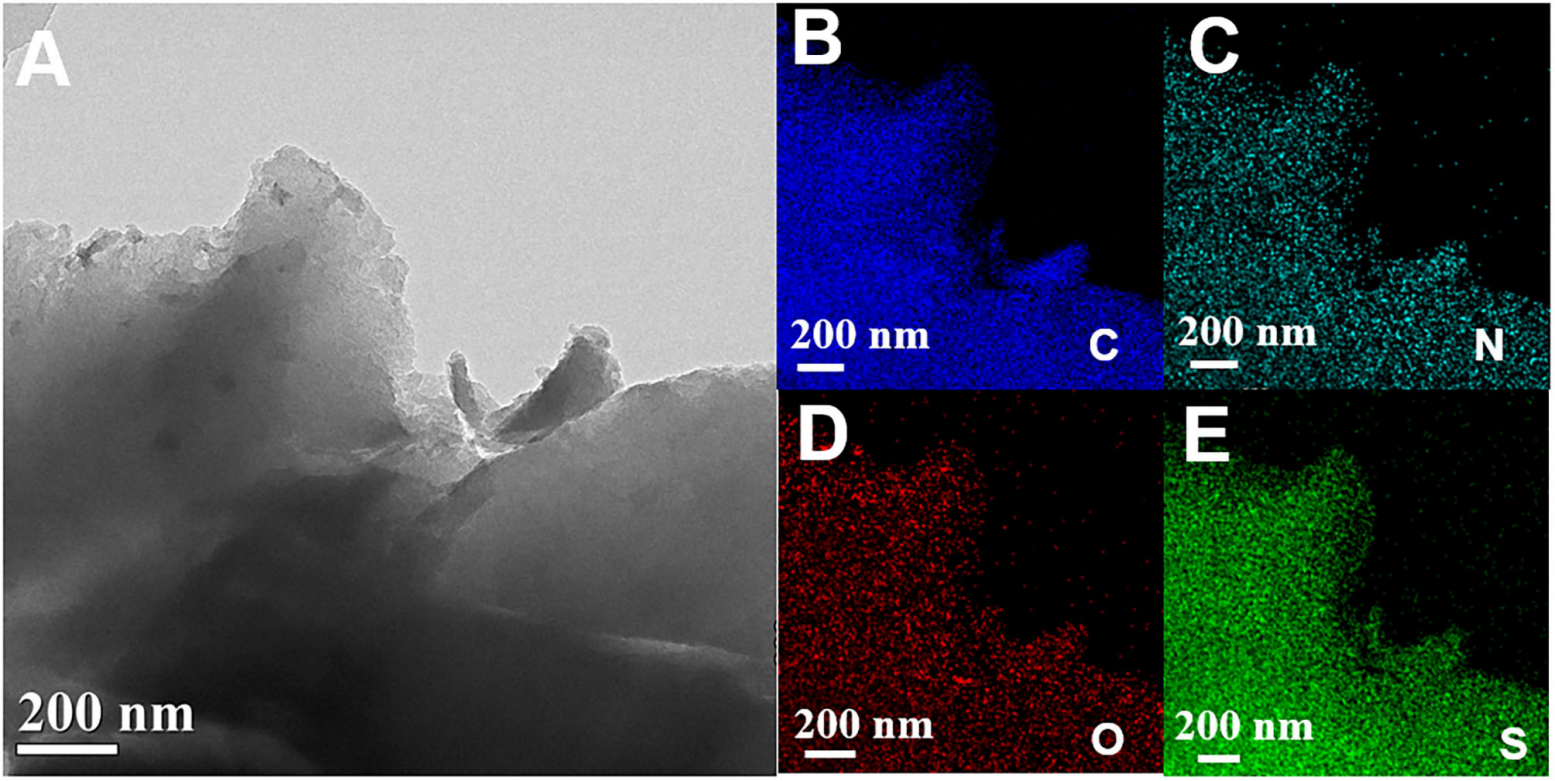
**(A)** TEM image, and **(B–E)** elemental mappings of CTF-PO71/S.

**Figure 5:**
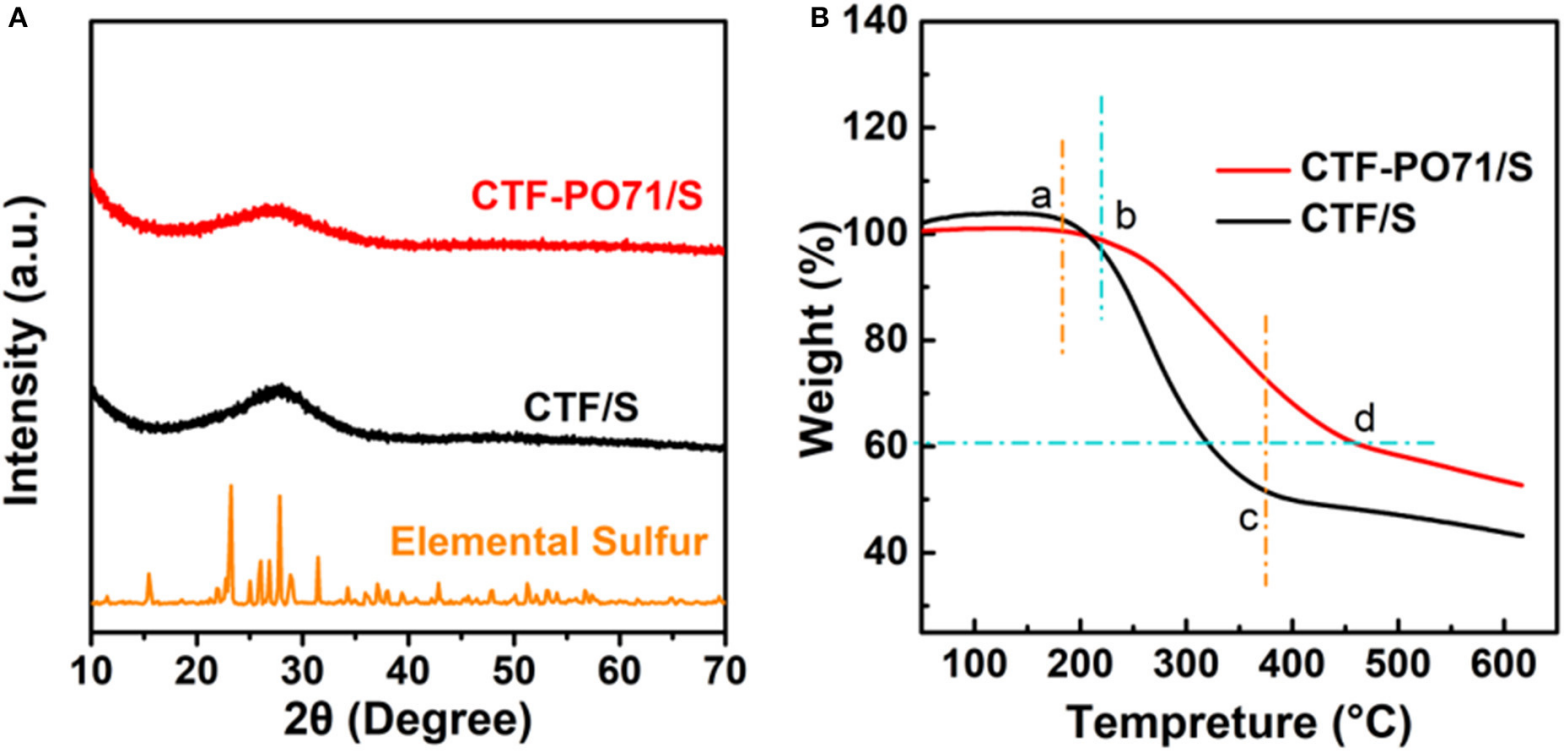
**(A)** PXRD patterns of CTF-PO71/S (red), CTF/S (dark), and elemental sulfur (orange). **(B)** TGA curves of CTF-PO71/S and CTF/S.

The CTF-PO71/S composites were synthesized through a typical melting–diffusion method (Supplementary Material). After sulfur impregnation, the layer structure of CTF-PO71 still retains the CTF-PO71/S composite (Figure 4A, Supplementary Figure 6A), suggesting the incorporation of sulfur in the nanopores of CTF-PO71 rather than on the surface. The mapping images (Figures 4B–E, Supplementary Figures 6B–D) demonstrate that sulfur was uniformly dispersed in the network of CTF-PO71 material. This could be further verified by XRD (Figure 5A), from which the crystalline sulfur peaks disappear in both CTF-PO71/S and CTF/S composites. These results illustrate the homogenous incorporation of sulfur into the confined nanopores with a highly dispersed amorphous state, thus permitting intimate contact between host and sulfur (Xu et al., 2019a). The sulfur loading was calculated by a difference in mass changes before and after melt diffusion and is ~50% (Supplementary Material). Thermal Gravimetric analysis (TGA) in Ar atmosphere was also conducted (Figure 5B). It has been verified from previous reports that pure CTF and CTF-PO71 could maintain good thermal stability below 500°C (Liao et al., 2014; Lu Y. et al., 2018). The massive weight loss of the two composites at lower temperatures can be attributed to escape of sulfur vapor. The weight loss region of CTF-PO71/S reveals a significant delay (b point) and afterwards a relatively smoother slope (b-d region) compared with that of CTF/S (a point and a–c), indicating much stronger interaction between the CTF-PO71 network and S (Xu et al., 2019b) (Supplementary Material).

**Figure 6:**
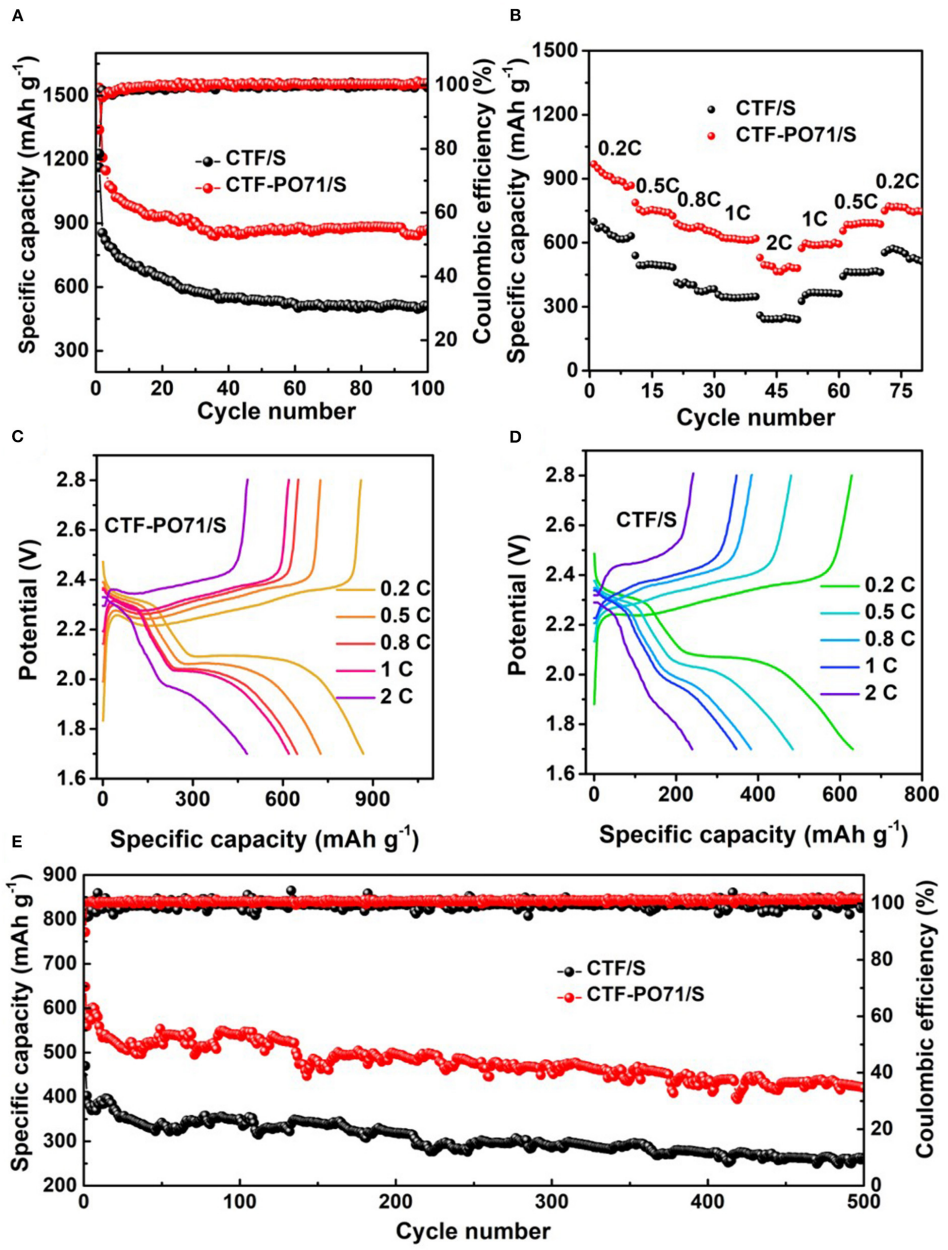
**(A)** Cycling performance and Coulombic efficiency of CTF/S and CTF-PO71/S at 0.1C. **(B)** Rate performance measured at various C rates with 10 cycles at each rate. Discharge and charge curves of **(C)** CTF-PO71/S and **(D)** CTF/S recorded at last cycle of each rate. **(E)** Long cycling performance and Coulombic efficiency of CTF/S and CTF-PO71/S at 1C.

The electrochemical performance of CTF-PO71/S and CTF/S as electrode materials was characterized in coin cells. To analyze the kinetics of electrochemical reaction, Supplementary Figure 7 shows the cyclic voltammogram (CV) curves of CTF-PO71/S electrode at a scan rate of 0.1 mV/s, where it exhibits typical characteristics for reaction of lithium with sulfur. During the first cycle of cathodic scan, two reduction peaks at 2.24 and 1.98 V correspond to the reduction of S_8_ to long-chain polysulfides followed by transformation of long-chain polysulfides to Li_2_S_2_ and/or Li_2_S, respectively (Xu et al., 2015; Jiang et al., 2018). The following oxidation process shows one peak at around 2.40 V attributed to the inverse process of polysulfide conversion back to elemental S. As the scan cycles increase, the positions of reduction peaks shift to higher voltage values while oxidation peak position shifts to lower voltage slightly, demonstrating the decrease of the polarization of reaction after first cycle of activation. This sulfur redox behavior can be also observed by the galvanostatic charge–discharge profiles (Supplementary Figure 8), which reveal two discharge plateaux and one charge quasi-plateau of CTF-PO71/S. The CTF-PO71 with developed pore structure and amide-based functionalities is beneficial for trapping polysulfides both physically and chemically, thereby exhibiting significant advantages on capacity and cycling stability compared with CTF. The electrochemical performances were thus measured first. The CTF-PO71/S composite delivers an initial discharge capacity of 1537 mAh g^−1^ at 0.1C (1378 mAh g^−1^ above 1.8 V, Figure 6A, Supplementary Figure 8) with the Coulombic efficiency (CE) of 86% and retains 869.9 mAh g^−1^ after 100 cycles (56.6% capacity retention), which are superior to that of CTF/S (1162 mAh g^−1^ at 0.1C, with CE of 78%, and retention of 43.9% after 100 cycles).

Significant advantages of CTF-PO71/S electrode are also seen in rate performance (Figures 6B–D), from which enhanced capacity of 868 mA h g^−1^, 726 mA h g^−1^, 648 mA h g^−1^, 619 mA h g^−1^, and 480 mA h g^−1^ at a current density of 0.2C, 0.5C, 0.8C, 1C, and 2C are obtained, and a retention of 749 mA h g^−1^ can be achieved when it recovers to 0.2C. The charge–discharge profiles for different cycles from 0.2C to 2C are also shown (Figures 6C,D), revealing clear distinct discharge and charge plateaus, aligning well with observations from the CV curves. Suppressing the capacity fading during long-term cycling is always a target goal for Li-S batteries. An exceptional long cycle performance was exhibited by CTF-PO71/S (Figure 6E). The capacity of 421 mAh g^−1^ remained at 1C after 500 cycles (65% capacity retention), which is obviously higher than the 261 mAh g^−1^ of the CTF/S electrode with a 56% capacity retention. The performances are comparable or even better than some previously reported porous polymer hosts (Supplementary Table 3). We also tested the material with a higher sulfur content of 67% in CTF-PO71 and CTF, and the result shows that CTF-PO71/S still outperform CTF/S, despite the overall decreased performances (Supplementary Figure 9). Much higher sulfur loading in the cathode electrode and lean electrolyte should be taken into account in the future for practical applications. CTF-PO71-1/S and CTF-PO71-10/S were also evaluated (Supplementary Figure 10), and CTF-PO71/S shows higher electrochemical performances to CTF-PO71-1/S and is comparable to CTF-PO71-10/S. This is probably the result of porosity and functionalities in CTF-PO71s under different amount of ZnCl_2_.

To clarify the strong interactions of CTF-PO71 with polysulfides, we carried out the adsorption test toward Li_2_S_6_ (Liang et al., 2015; Xie et al., 2017). A complete decoloration was observed when immersing CTF-PO71 into Li_2_S_6_ solution after 2 h, whereas the solution remains light yellow using CFT as a control (Supplementary Figure 11A). This result indicates a strong adsorptive capability of CTF-PO71 toward Li_2_S_6_ resulted from the more developed pore structure via physical adsorption and functionalities via chemisorption. Such chemical interactions were further corroborated by analyzing XPS of CTF-PO71 after adsorption (abbreviated as PS@CTF-PO71) (Supplementary Figure 11B). Li_2_S_6_ exhibits two sets of double peaks, corresponding to the terminal S (S_T_) and bridge S (S_B_). Due to the interaction of Li_2_S_6_ with triazine framework, both samples show positive shift in S_T_ and S_B_ peaks. The S_T_/S_B_ ratio of Li_2_S_6_ is 0.57, which is slightly higher than the theoretical value of 0.5 due to the oxidation of sample during transferring and testing process (Liang et al., 2015). It was reported that C=O functionality could interact with polysulfides (Song et al., 2014; Chen et al., 2015; Li G. et al., 2018; Xu et al., 2019c). PS@CTF-PO71 shows much lower S_T_/S_B_ ratio (0.29), as compared to Li_2_S_6_ and PS@CTF, validating the interaction of amide in CTF-PO71 with polysulfide.

### Organic Dye Adsorption Performance

Numerous organic dyes are widely used in textiles, paints, plastics, etc., creating one of the most serious environmental pollution sources nowadays with the rapid development of industrialization (Wang T. et al., 2014). Various methods have been applied for the removal of the dye molecules, among which the adsorption method by introducing suitable adsorbent is considered to be most efficient, economic and environmental-friendly (Yu S. et al., 2018). CTF-PO71s are promising as adsorbent benefiting from the high surface areas with tremendous nanopores and amide-based functionalities.

**Figure 7:**
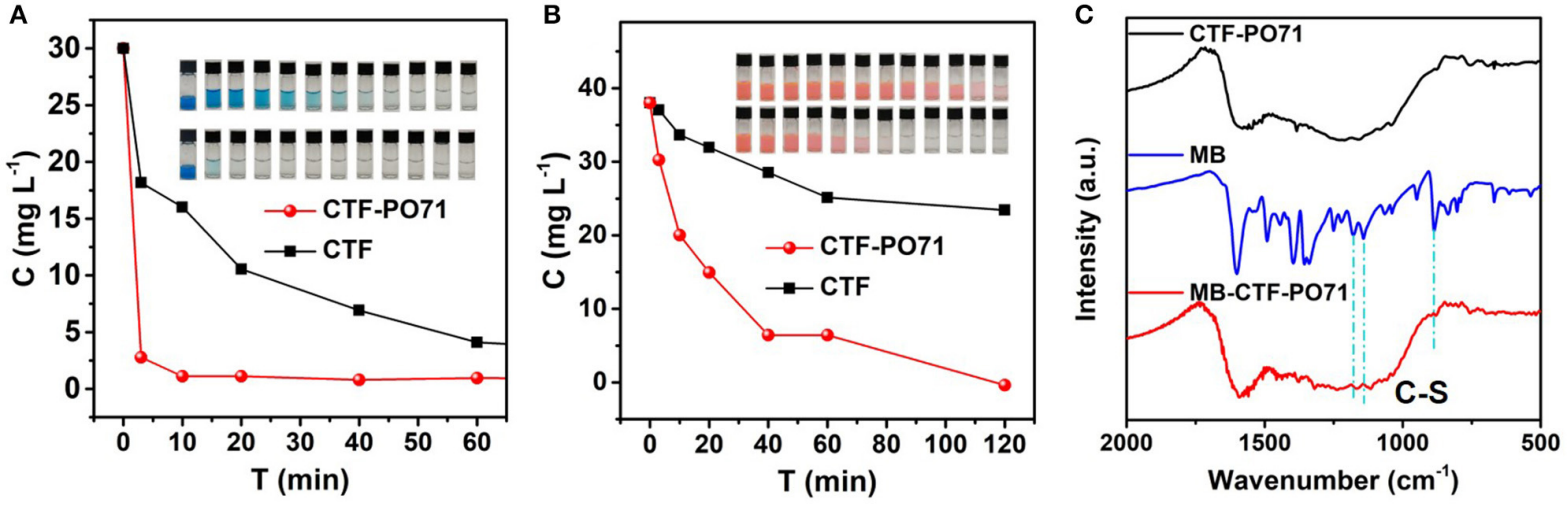
Concentrations of **(A)** MB and **(B)** R6G solution as a function of adsorption time with CTF-PO71 and CTF, and the inset showing the digital photos of dye solution after various adsorption times of 0 min, 3 min, 10 min, 20 min, 40 min, 60 min, 2 h, 4 h, 6 h, 8 h, 18 h, and 24 h. **(C)** FT-IR spectra of CTF-PO71, MB, and the sample MB-CTF-PO71 after adsorption of MB.

The organic dye adsorption performance of CTF-PO71 and CTF was studied by introducing organic dyes of methylene blue (MB) and rhodamine 6G (R6G) as adsorbates. Figure 7A shows the adsorption performances and the corresponding digital photos of MB solution after adsorption. Compared with unfunctional CTF, CTF-PO71 exhibits a significantly better performance, and the solution becomes clear within 5 min (Figure 7A). Similarly, CTF-PO71 also shows superior adsorption behavior compared to CTF for R6G (Figure 7B). This is on one hand due to the higher surface area of CTF-PO71. Moreover, there exists chemical interactions between the dye molecules and CTF-PO71, as revealed by FT-IR spectra after adsorption of MB (abbreviated by MB-CTF and MB-CTF-PO71) (Figure 7C and Supplementary Figure 12). The C-S stretching vibrations from MB, represented by a double band positioned at 1178 and 1142 cm^−1^ shift to 1166 and 1117 cm^−1^ in MB-CTF-PO71, respectively. In contrast, the spectra of MB-CTF does not show similar peaks shift (Yu W. et al., 2018). These results suggest the chemical interactions of amide group in CTF-PO71 with MB.

**Figure 8:**
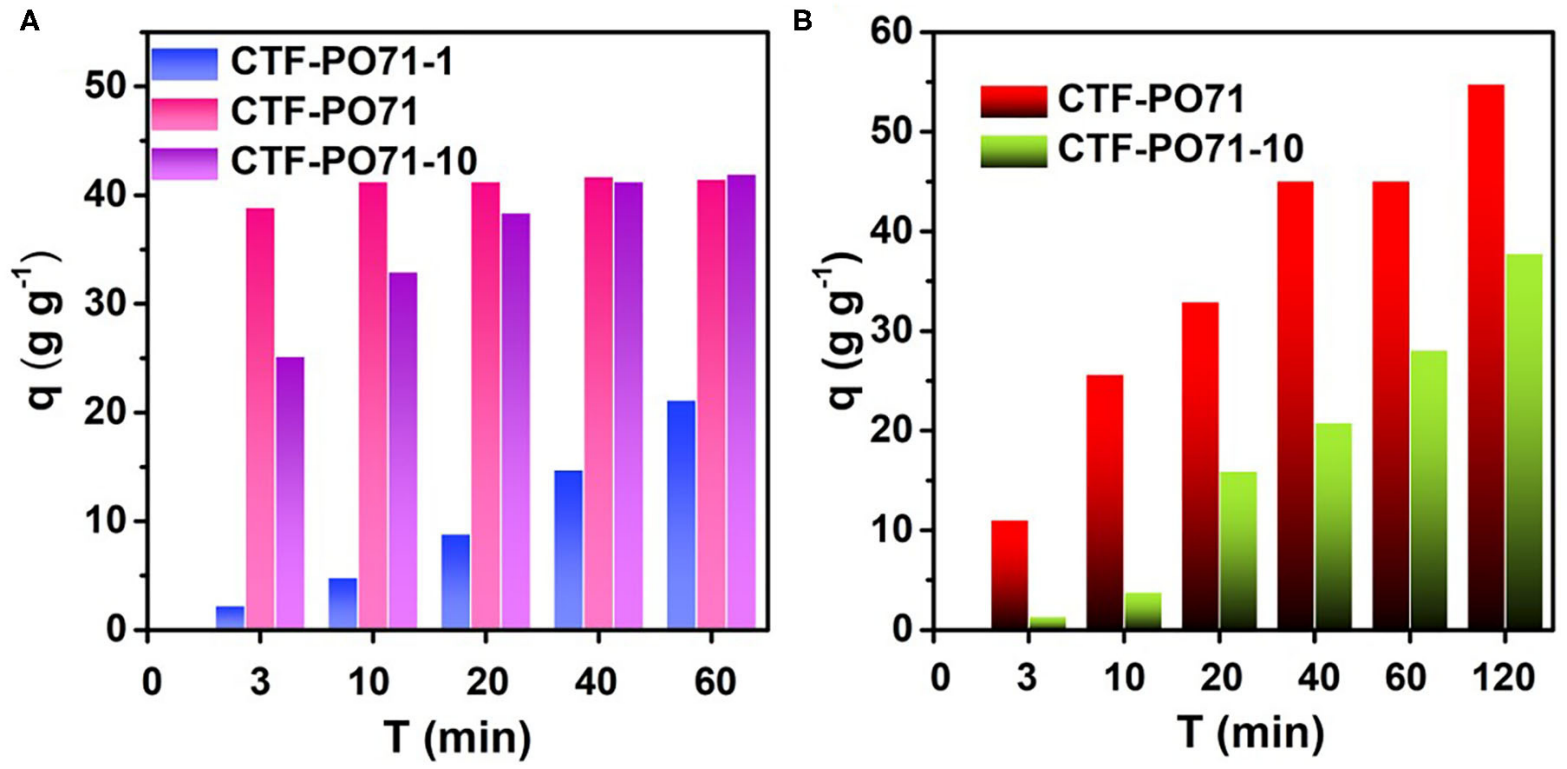
Adsorption capacities on **(A)** MB and **(B)** R6G of CTF-PO71s within a series of adsorption time.

**Figure 9:**
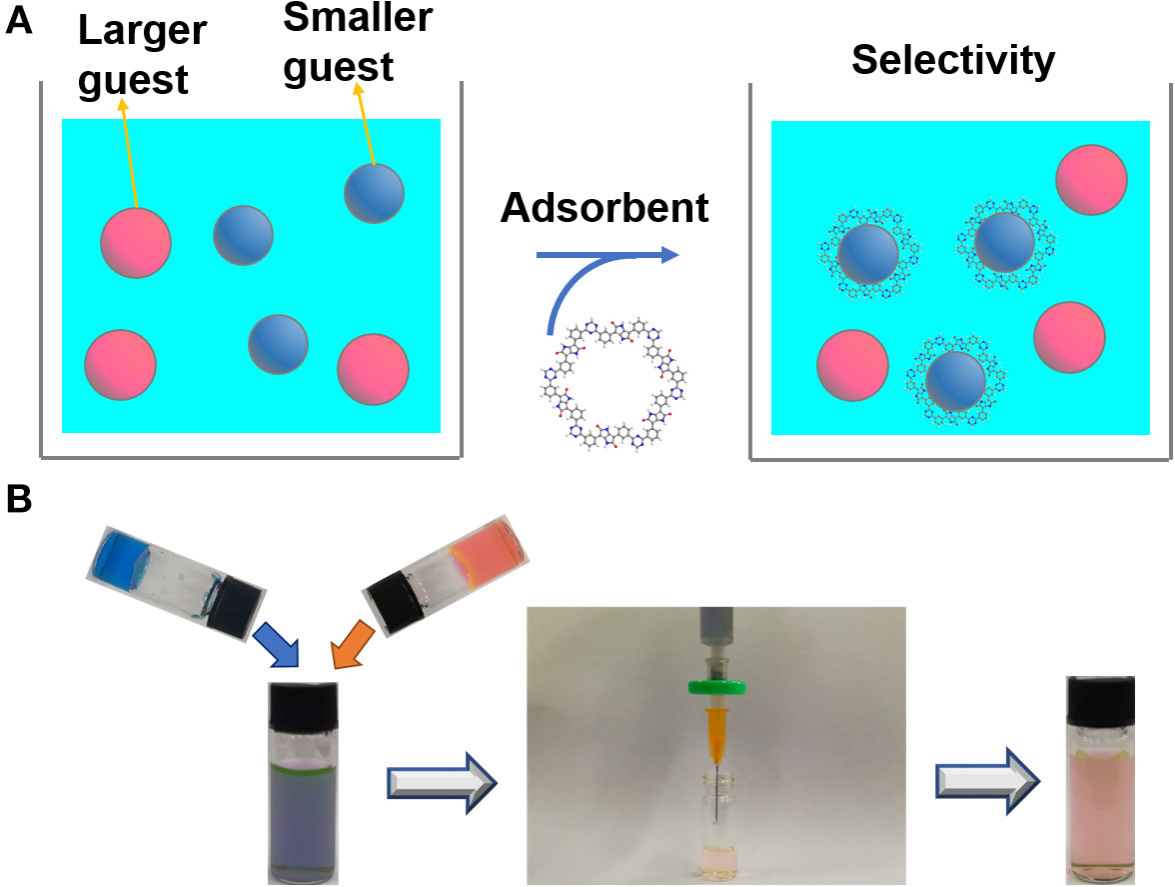
**(A)** Schematic illustration of selective adsorption. **(B)** Selective adsorption experiments of mix solution of MB and R6G using CTF-PO71-1 in a syringe.

The adsorption behavior of other CTF-PO71s was further evaluated. Figure 8A and Supplementary Figure 13 show the adsorption performance of CTF-PO71s toward MB. CTF-PO71 exhibits highest adsorption capacities. For CTF-PO71-1, the low performance might be due to its narrow pore distribution of 0.61 nm with the lowest mesopore ratio (Supplementary Table 1), which provides less opportunity for fast access of MB molecules (1.43 × 0.61 × 0.40 nm, Supplementary Table 4) inside the polymer network (Li et al., 2014). For CTF-PO71-10, although it shows a much higher surface area and mesopore ratio than CTF-PO71, the performance is still lower, which is mainly attributed to the decreased amide functionalities under higher amount of ZnCl_2_. Such behavior becomes more pronounced when using dye R6G bearing a larger molecular size (Supplementary Table 4) as adsorbate (Figure 8B), where CTF-PO71-1 exhibits almost little adsorption toward R6G (Supplementary Figure 14) due to the size exclusion effect of R6G to the micropores of CTF-PO71-1 (Gupta and Khatri, 2019). Dynamic adsorption experiments were further performed to better understand the size exclusion effect (Figure 9A). Selective adsorption was observed in the dynamic adsorption process of mixed solution of MB and R6G (Figure 9B). After passing through a syringe, the purple color of the mixed MB/R6G solution turns into pink of R6G, confirming the selective adsorption of MB. Such selective adsorption behavior offers an opportunity for potential application of molecule separation.

## Conclusion

In conclusion, we presented a series of amide functionalized CTF-PO71s and investigated the effect of pore structure and functional groups on capture-based processes, including impregnation of sulfur as host materials in lithium-sulfur batteries and liquid-phase dye adsorption. As a sulfur host, the significant improvement of electrochemical performance was achieved for functionalized CTF-PO71 compared with that of nonfunctionalized CTF. The CTF-PO71/S shows high capacity of 1537 mAh g^−1^ at 0.1C, stable cycling performance (56.6% capacity retention after 100 cycles at 0.1C), and good rate performance (480 mAh g^−1^ at 2C). The promoted entrapment of polysulfide results from the cooperative effect of physical confinement by high porosity of the frameworks and the chemisorption resulting from functional groups in CTF-PO71 networks. The organic dye adsorption performance demonstrates the important role of the amide functional group for chemical interaction and the pore structure for facile access of dye molecules, thereby significantly promoting the adsorption performance.

## Data Availability Statement

The original contributions presented in the study are included in the article/Supplementary Material, further inquiries can be directed to the corresponding author/s.

## Author Contributions

FX and HW conceived the project and supervised the research works. QL and SY carried out the synthesis. SY, QL, YZ, and XX contributed to structural characterization and electrochemical characterization. QL performed experiments of dye solution adsorption. HL and YL provided advice for the research. FX and QL wrote the manuscript. All authors contributed to the article and approved the submitted version.

## Conflict of Interest

The authors declare that the research was conducted in the absence of any commercial or financial relationships that could be construed as a potential conflict of interest.
