# Supplementary material for: Porous Functionalized Covalent-Triazine Frameworks for Enhanced Adsorption Toward Polysulfides in Li-S Batteries and Organic Dyes
Source: Front Chem. 2020 Dec 2;8:584204. doi: 10.3389/fchem.2020.584204 (PMC7738625; doi:10.3389/fchem.2020.584204)
Supplement: Supplementary file 1 [file Data_Sheet_1.pdf]

Supplementary Material

1 Materials

Zinc chloride (anhydrous, 98%) and lithium sulfide (Li_2S) were purchased from Alfa Aesar and stored in a glove box and used as received. 1,4-dicyanobenzene (DCB) (98%), and sublimated sulfur (99.99%) were purchased from Aladdin. Pigment orange 71 (PO71) was purchased from Shanghai OurChem BioTechnology Co., Ltd and used without further purification. Methylene Blue trihydrate (MB), Tetrahydrofuran (THF) and HCl were purchased from Sinopharm Chemical Reagent Co., Ltd. Polyvinylidene fluoride binder (PVDF), conductive carbon black (Super P Li), and N-methyl-2-pyrrolidinone (NMP) (99.90%) were purchased from MTI-KEJING. 1,3-dioxolane (DOL) and 1,2-dimethoxyethane (DME) were purchased from Aladdin. Rhodamine 6G (95%) was purchased from Energy Chemical.Supplementary material is not typeset so please ensure that all information is clearly presented, the appropriate caption is included in the file and not in the manuscript, and that the style conforms to the rest of the article. To avoid discrepancies between the published article and the supplementary material, please do not add the title, author list, affiliations or correspondence in the supplementary files.

2 Preparation of CTF-PO71/S and CTF/S

The CTF-PO71/S and CTF/S composites were synthesized through melting-diffusion method. The CTF-PO71 and elemental sulfur were mixed and grinded uniformly and then transferred into a vial, and sealed after being purged with N_2 for 15 min. The fabrication process was taken place in a tube oven, where the mixture in the vial was heated to 115 °C firstly with a rate of 1 °C min^{-1}. Then the temperature was increased to 155 °C with a rate of 0.5 °C min^{-1} and remained for 12 h to allow the melted sulfur to fully diffuse into the nanopores of host materials. After cooling down to room temperature, the CTF-PO71/S and CTF/S composites were thereby obtained.

3 Characterization

3.1 Material Characterization

The micromorphology was measured with FEI Nova Nanonova 450 scanning electron microscope, and the elemental mapping of materials was performed with Tecnai G2 SpiritT transmission Electron Microscopy, equipped with energy dispersive X-ray Spectroscopy. Chemical structure of materials was investigated by Nicolet iS50 Fourier transform infrared (FT-IR) with KBr pellet. Raman spectra was conducted by Renishaw inVia laser-micro-Raman spectra. X-ray photoelectron spectroscopy (XPS) measurement for CTF, CTF-PO71s, CTF/S, CTF-PO71s/S, MB-CTF, and MB-CTF-PO71s was performed by Thermo SCIENTIFIC ESCALAB 250Xi XPS (Al K radiation). The crystallinity the X-ray powder diffraction (XRD) was carried out with X'Pert PRO MPD X-ray power diffractometer (Cu-Kα radiation, λ = 1.54056) under room temperature. The scanning was in the range of 2 theta = 10 to 90° with a scan rate of 5° min^{-1}. The porous structure of materials was analyzed through N_2 adsorption-desorption test by Micromeritics ASAP 2020 surface area analyzer under 77 K. The specific surface area (S_{BET}) of samples were calculated by Brunauer-Emmett-Teller

(BET) method. And pore size distribution was analyzed by nonlocal density function theory (NLDFT) method. The micropore volume (V_{mic}) was measured by t-plot method and total pore volume (V_{total}) was obtained by single point adsorption amount at P/P_0 of 0.99. Mesopore ratio was obtained by the mesopore volume from NLDFT divided by accumulated DFT pore volume.

3.2 Elucidation on TGA curves and determination of sulfur content

It was shown that the sulfur loss in CTF/S composite starts from 183 and ends at 376 °C, and the total mass loss reveals a 50 wt% sulfur loading of the composite. In contrast, the weight loss region of CTF-PO71/S reveals a significant delay that starts at higher temperature of 226 °C and a less obvious turning point appears at 465 °C, indicating much stronger interaction between the CTF-PO71 network and S. Furthermore, such stronger interaction could also be demonstrated from smoother slope of the CTF-PO71/S curve in *b*-*d* region than that in analogous *a*-*c* region of the CTF/S curve. It should be noted that the point *d* (465 °C, 40% weight loss) may not be the definitive terminal point of sulfur loss, since the remaining curve still follows a quick downward trend. Besides, the sulfur loading for CTF-PO71/S is calculated by method of subtractive: $S_{loading}\%= [Ms-(M_{total-bef}-M_{total-aft})] /M_{total-aft}$, in which M_s refers to the initial addition mass of the sulfur, and $M_{total-bef}$ and $M_{total-aft}$ refer to the mass of the mixture before and after the melt diffusion process, respectively.

3.3 Li_2S_6 adsorption test

Preparation of Li_2S_6 solution and the adsorption test were carried out in an argon-filled glove box. The Li_2S_6 solution (2 mmol L^{-1}) was prepared by dissolving Li_2S and elemental sulfur with a molar ratio of 1:5 in DME&DOL (1:1 v/ v) with vigorous stirring at 70 ℃ to form a transparent yellow solution. 15 mg of CTF-PO71 or CTF was added into a 5 mL glass vials of which contains 4 mL of the above Li_2S_6 solution. Photos were taken by static adsorption for 2 h, whereas a pristine Li_2S_6 solution was used for comparison.

4 Supplementary Figures and Tables

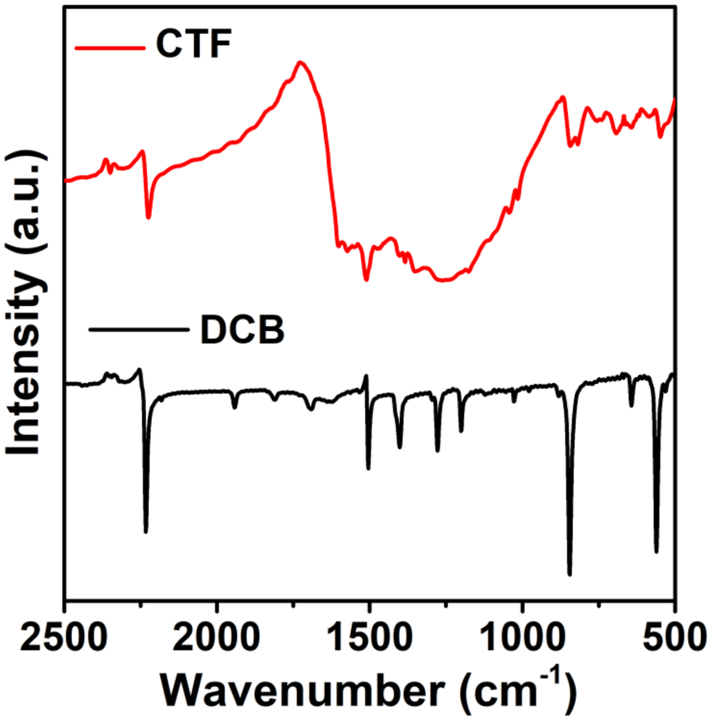

Figure S1. FT-IR spectra of CTF and DCB.

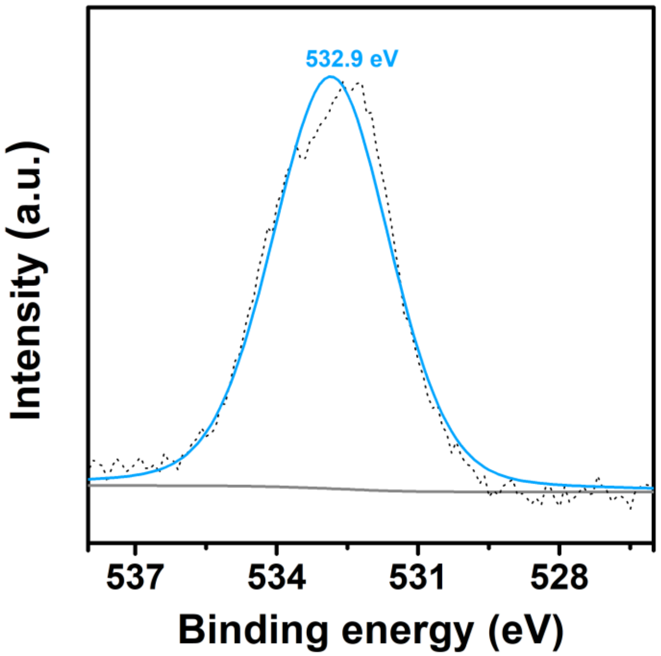

Figure S2. O1s spectrum of CTF-PO71.

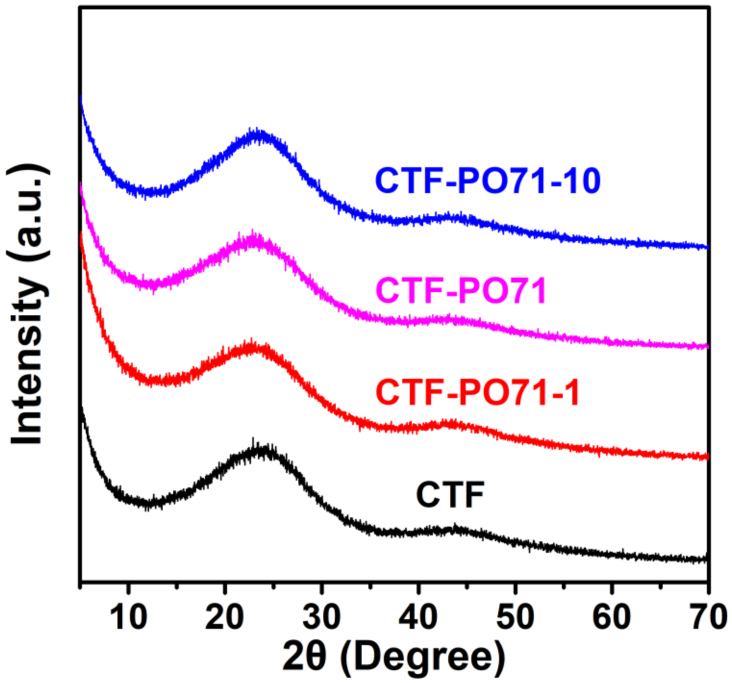

Figure S3. PXRD patterns of CTF-PO71s and CTF.

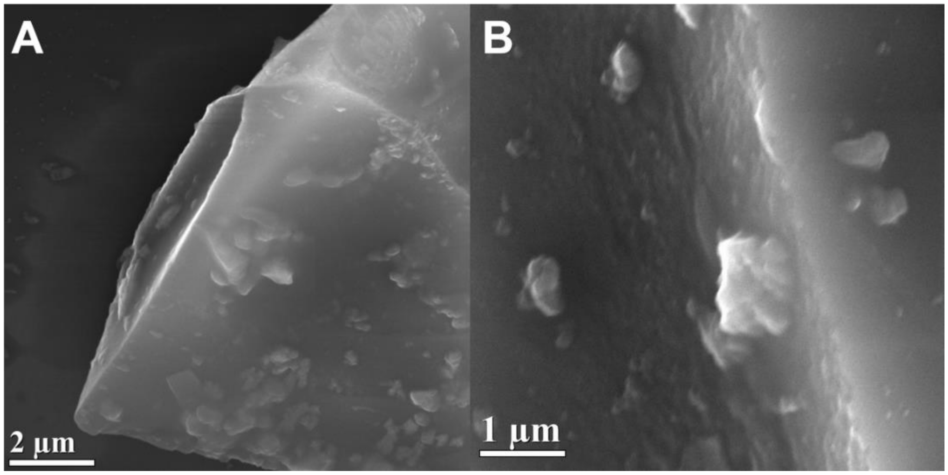

Figure S4. (A) and (B) SEM images of CTF-PO71.

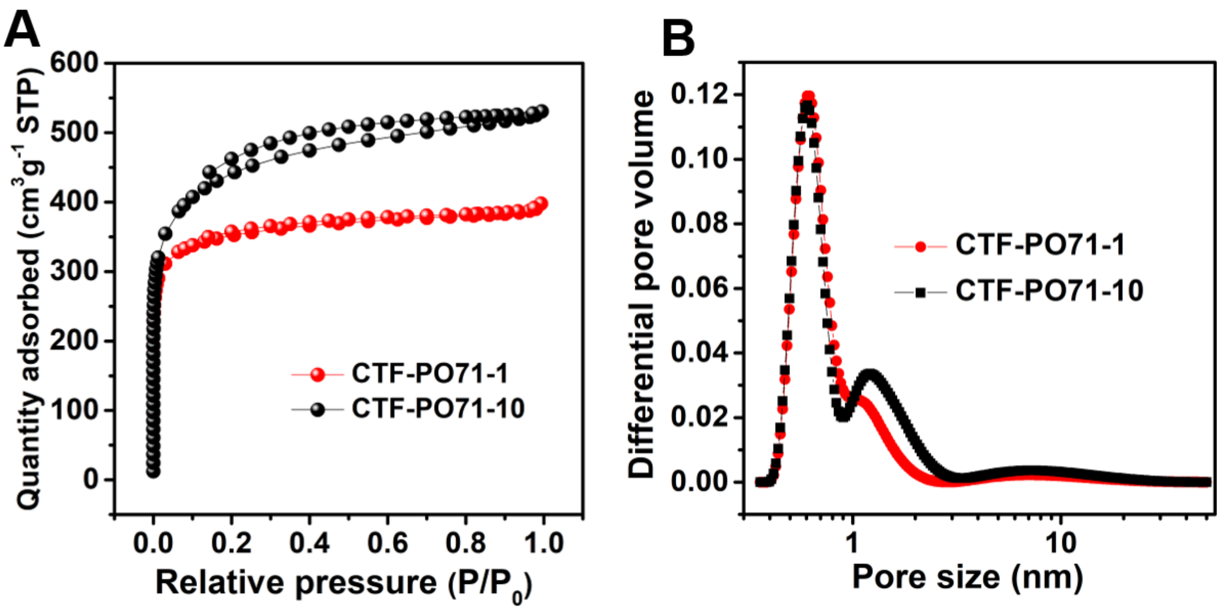

Figure S5. (A) Nitrogen adsorption-desorption isotherms of CTF-PO71s. (B) Pore size analysis with NLDFT model of CTF-PO71s.

Table S1. Summary of the pore parameters for CTF and CTF-PO71s.

**Sample**	S_{BET} ($m^2\ g^{-1}$)	V_{mic} ($cm^3\ g^{-1}$)	V_{total} ($cm^3\ g^{-1}$)	**Mesopore ratio (%)**
CTF-PO71-1	1185	0.41	0.62	10.3 %
CTF-PO71	1001	0.27	0.54	22.7 %
CTF-PO71-10	1519	0.36	0.82	25.8 %
CTF	746	0.22	0.38	17.0 %

Table S2. Elemental composition determined by XPS of CTF-PO71s.

Sample	Content			
	**C**	**N**	**O**	**N/C**
CTF-PO71 (theoretical value)	55.6%	11.1%	5.56%	0.2
CTF-PO71-1	79.78%	8.95%	11.26%	0.11
CTF-PO71	80.82%	6.23%	12.96%	0.08
CTF-PO71-10	80.10%	4.81%	15.09%	0.06

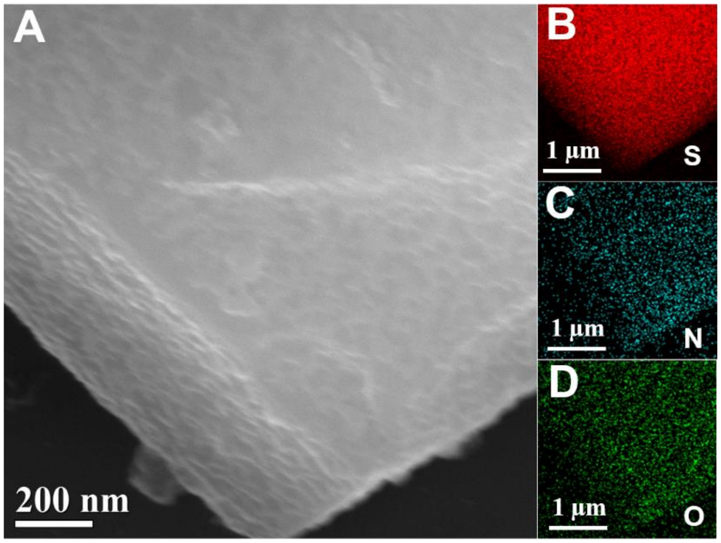

Figure S6. SEM images of (A) CTF-PO71/S and EDS mapping of (B) sulfur, (C) nitrogen, (D) oxygen.

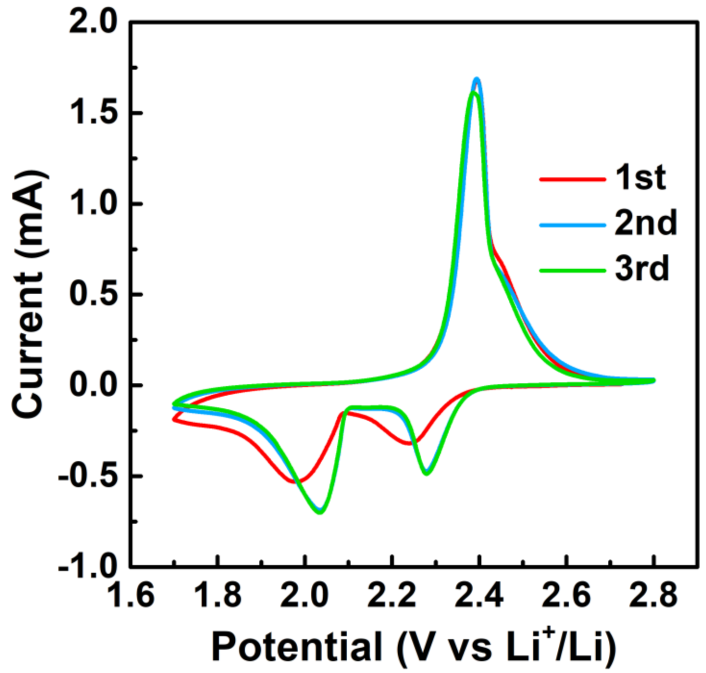

Figure S7. CV curves of the first 3 cycles for CTF-PO71/S at scan rate of 0.1 mV s^{-1}.

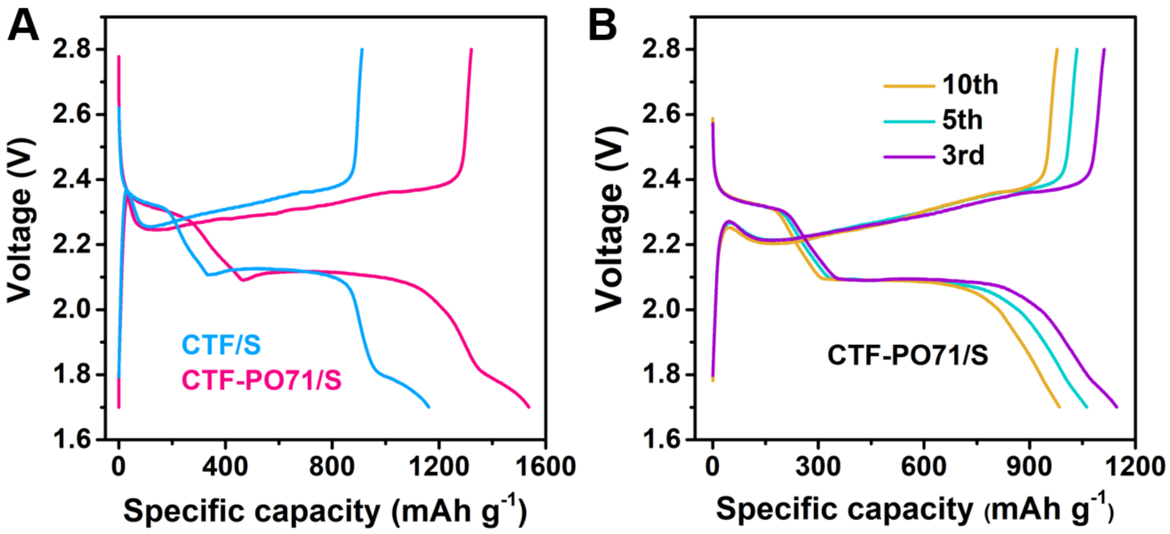

Figure S8. (A) First discharge and charge curves of CTF/S and CTF-PO71/S measured at 0.1 C. (B) The discharge and charge curve of CTF-PO71/S in 3rd, 5th, and 10th cycles at 0.1 C. The first discharge-charge curves show quasi plateau below 1.8 V and then disappear after 3 cycles. Thus, the first cycle with quasi plateau below 1.8 V could be related to the decomposition of $LiNO_3$ for the formation of stable SEI film.

Table S3. Summary of some reported Li-S battery performances using porous organic polymer hosts.

Host material	Capacitance /mAh g^{-1} (Rate/ C)	Cycle stability / mAh g^{-1} (cycle number, Rate/ C)	Rate performance (retention/ % (Rate/ C))	Initial coulombic efficiency/ % (Rate/ C)	Voltage range/ V	Sulfur loading / wt.%	Ref.
CTF-PO71	1537 (0.1)	421 (500, 1)	55 (0.2~2)	86 (0.1)	1,7~2.8	50	This work
TAPB-PDA-COF	1357 (0.2)	705 (210, 0.2)	53.85 (0.2~1)		1.8~2.8	60	J Energy Chem., 2019, 28, 54
TFPPy–ETTA–COF	1069 (0.1)	~577 (130, 0.1)	49.7 (0.2~1)		1.7~2.8		Chem. Sci., 2019, 10, 6001
COF-F	1120 (0.1)	645 (100, 0.1)	~40 (0.1~2)		1.7~2.8	60	ACS Appl. Mater. Interfaces, 2018, 10, 42233
S-CTF-1	482.2 (1)	413.7 (300, 1)	59.8 (0.05~2)	94.4 (0.05)	1.7~2.7	62	Angew. Chem. Int. Ed. 2016, 55, 3106
COF-ETTA-ETTCA	1617 (0.1)	605 (528, 0.5)	11 (0.1~5)	98 (0.5)	1.8~2.8	88.4	ACS Appl. Mater. Interfaces 2020, 12, 34990
S-FCTF-400	741 (0.5)	494 (200, 0.5)	57 (0.2~2)		1.7~2.8	50	Energy Technol. 2019, 1900583
CTF-1	1197 (0.1)	762 (50, 0.1)	59 (0.1~1)	97 (0.2)	1.1~3.0	34	J. Mater. Chem. A, 2014, 2, 8854
FCTF-S	1296 (0.1)	833 (150, 0.5)		~98 (1)	1.7~2.8	51	ACS Appl. Mater. Interfaces 2017, 9, 3773

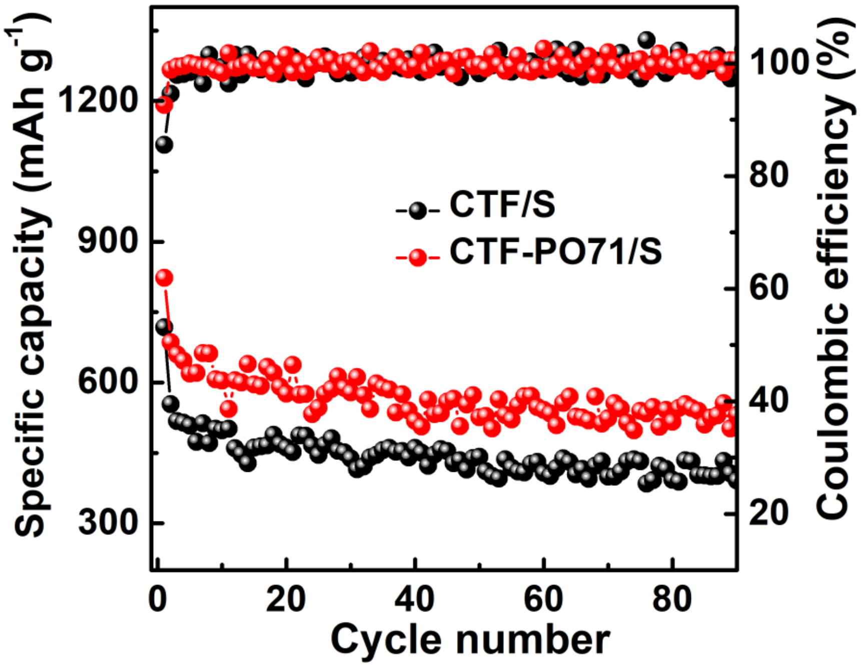

Figure S9. Cycling performances of CTF/S and CTF-PO71/S at 0.1 C with a higher sulfur content of 67%.

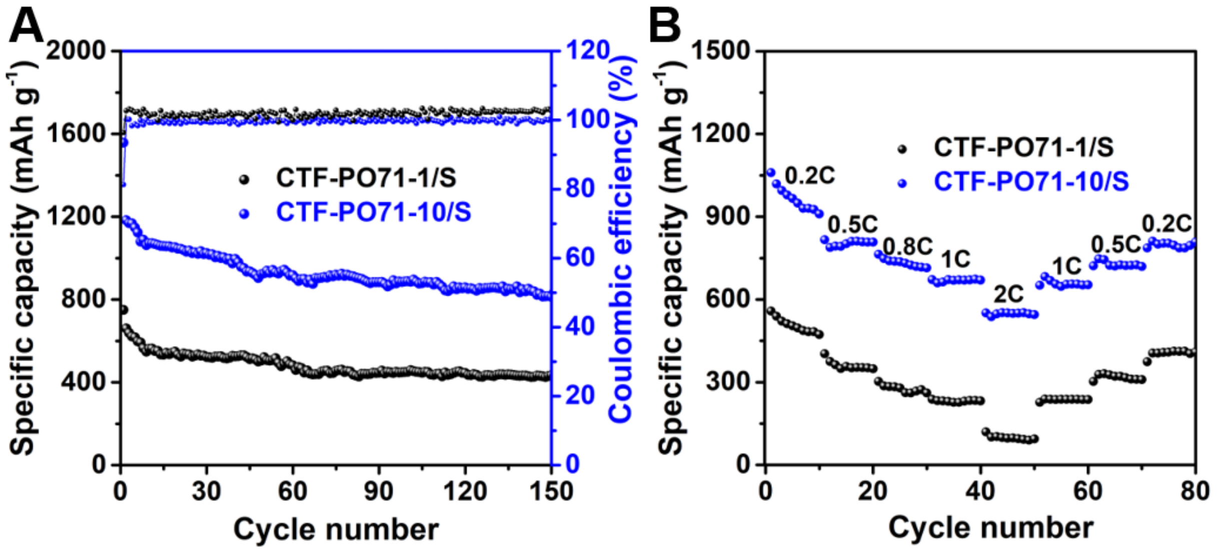

Figure S10. Cycling performances of CTF-PO71-1/S and CTF-PO71-10/S at (A) 0.1 C and (B) rate performances.

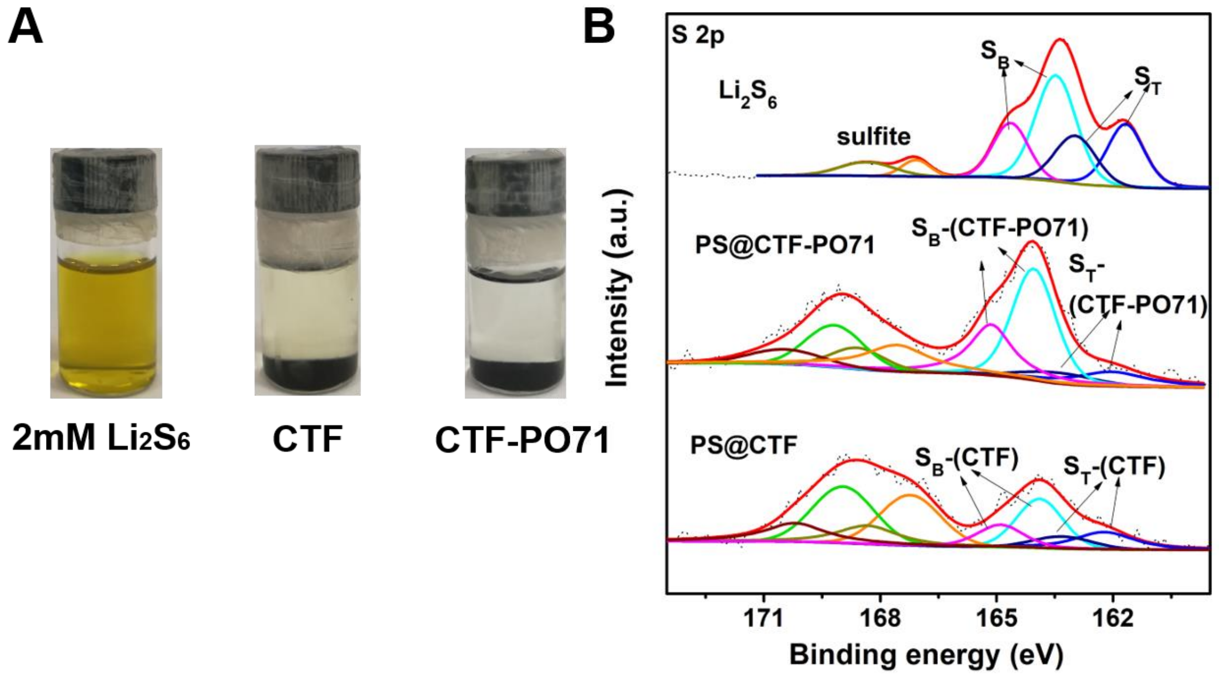

Figure S11. (A) Digital photos of original Li_2S_6 solution in DOL/DME (v/v=1:1) and the ones after being adsorbed by CTF and CTF-PO71 with 2 hours. (B) S2p spectra of Li_2S_6, CTF and CTF-PO71 after 2-hour adsorption of the Li_2S_6.

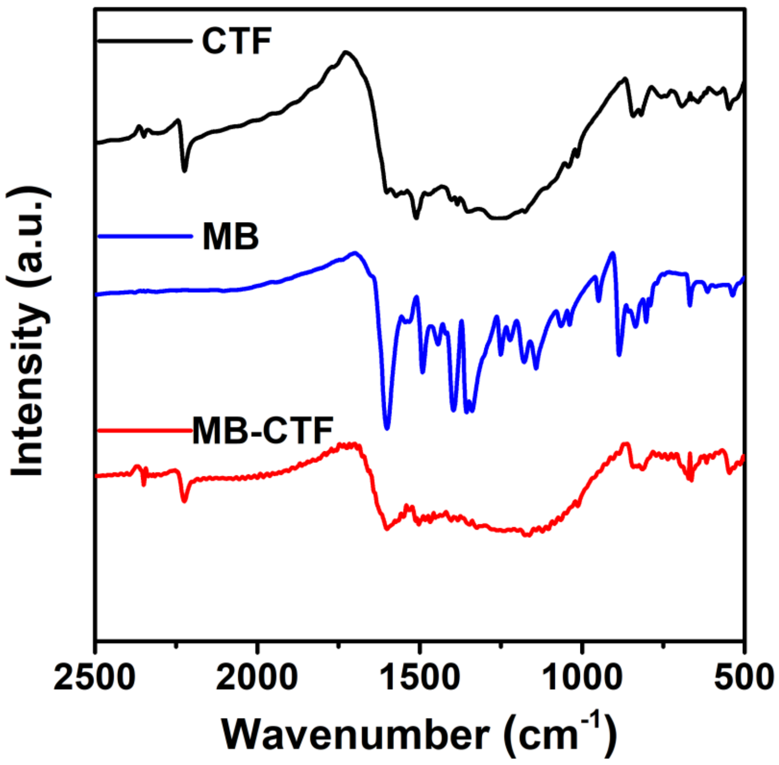

Figure S12. FT-IR spectra of CTF, MB and MB-CTF.

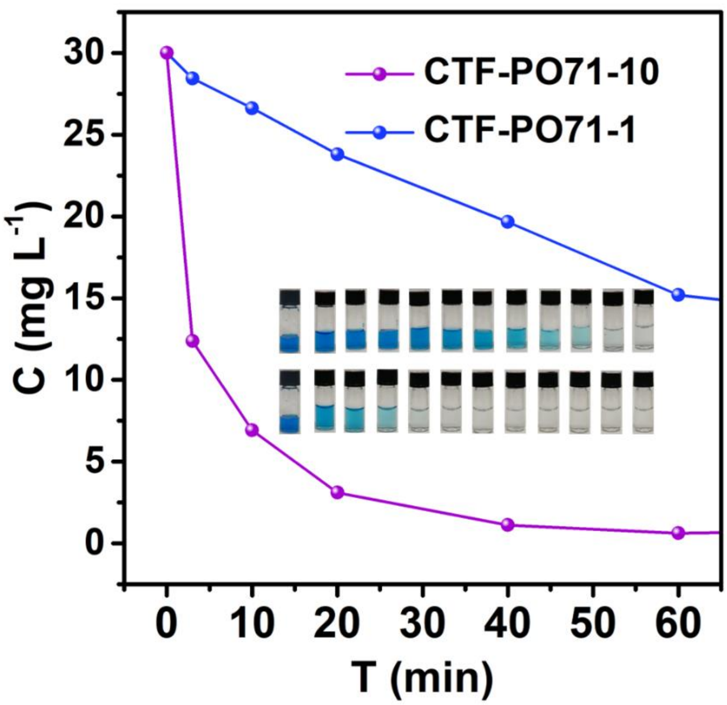

Figure S13. The MB concentration as a function of adsorption time by CTF-PO71-1 and CTF-PO71-10, and the inset showing the digital photos of dye solution after various adsorption times of 0 min, 3 min, 10 min, 20 min, 40 min, 60 min, 2 h, 4 h, 6 h, 8 h, 18 h and 24 h.

Table S4. Summary of the parameters of organic dyes.

Organic dyes	Formula	3-dimensional structure	MW (g mol^{-1})	Molecular size (nm)
**MB**	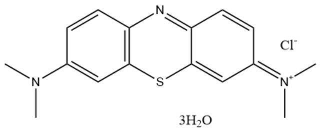	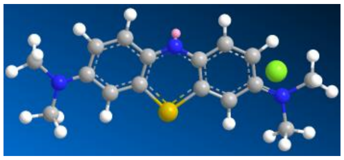	320	~1.4×0.6×0.4
**R6G**	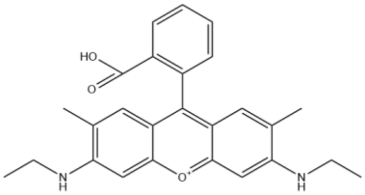	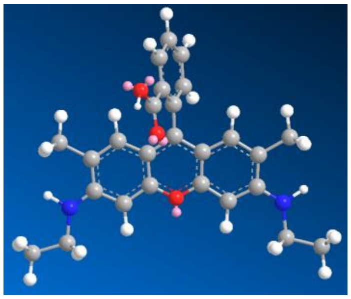	479	~1.3×0.6×1.2

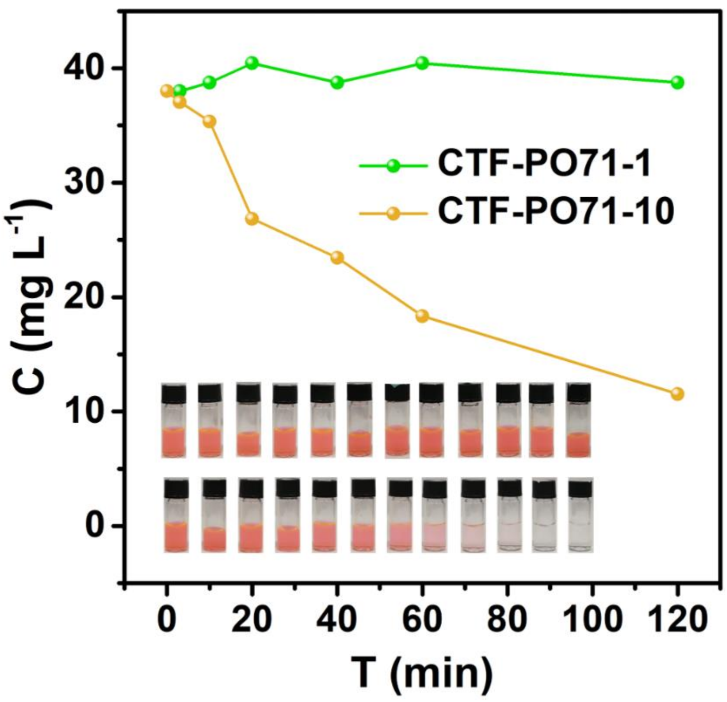

Figure S14. The R6G concentration as a function of adsorption time by CTF-PO71-1 and CTF-PO71-10, and the inset showing the digital photos of dye solution after various adsorption times of 0 min, 3 min, 10 min, 20 min, 40 min, 60 min, 2 h, 4 h, 6 h, 8 h, 18 h and 24 h.
